# Supplementary material for: Generating brain-wide connectome using synthetic axonal morphologies
Source: Nat Commun. 2025 Jul 18;16:6611. doi: 10.1038/s41467-025-62030-3 (PMC12271523; doi:10.1038/s41467-025-62030-3)
Supplement: Supplementary file 1 — Supplementary Information [file 41467_2025_62030_MOESM1_ESM.pdf]

10 Supplementary Materials

10.1 Acronyms

A list of brain regions acronyms used can be found in table S1.

Acronym	Name
ACA	Anterior cingulate area
ACAd5	Anterior cingulate area, dorsal part, layer 5
ACAd6a	Anterior cingulate area, dorsal part, layer 6a
ACAv5	Anterior cingulate area, ventral part, layer 5
AD	Anterodorsal nucleus
AI	Agranular insular area
AId5	Agranular insular area, dorsal part, layer 5
AId6a	Agranular insular area, dorsal part, layer 6a
AIp5	Agranular insular area, posterior part, layer 5
AIv5	Agranular insular area, ventral part, layer 5
AM	Anteromedial nucleus
APr	Area prostriata
AUD	Auditory areas
AUDd5	Dorsal auditory area, layer 5
AUDp5	Primary auditory area, layer 5
AUDpo5	Posterior auditory area, layer 5
AUDv5	Ventral auditory area, layer 5
AV	Anteroventral nucleus of thalamus
BST	Bed nuclei of the stria terminalis
CA	Ammon's horn
CL	Central lateral nucleus of the thalamus
CP	Caudoputamen
CTXpl	Cortical plate
CTXsp	Cortical subplate
DG	Dentate gyrus
DMH	Dorsomedial nucleus of the hypothalamus
ECT	Ectorhinal area
ECT5	Ectorhinal area, layer 5
ECT6a	Ectorhinal area, layer 6a
ENT	Entorhinal area
Eth	Ethmoid nucleus of the thalamus
FRP	Frontal pole, cerebral cortex
FRP5	Frontal pole, layer 5
GPi	Globus pallidus, internal segment
GU	Gustatory areas
GU5	Gustatory areas, layer 5
HPF	Hippocampal formation
HY	Hypothalamus

Acronym	Name
IAD	Interanterodorsal nucleus of the thalamus
IAM	Interanteromedial nucleus of the thalamus
IGL	Intergeniculate leaflet of the lateral geniculate complex
ILA	Infralimbic area
ILA5	Infralimbic area, layer 5
Isocortex	Isocortex
LD	Lateral dorsal nucleus of thalamus
LGd	Dorsal part of the lateral geniculate complex
LGv	Ventral part of the lateral geniculate complex
LH	Lateral habenula
LHA	Lateral hypothalamic area
LM	Lateral mammillary nucleus
LP	Lateral posterior nucleus of the thalamus
MB	Midbrain
MD	Mediodorsal nucleus of thalamus
MG	Medial geniculate complex
MH	Medial habenula
MM	Medial mammillary nucleus
MOp	Primary motor area
MOp1	Primary motor area, layer 1
MOp2	Primary motor area, layer 2
MOp3	Primary motor area, layer 3
MOp5	Primary motor area, layer 5
MOp6a	Primary motor area, layer 6a
MOp6b	Primary motor area, layer 6b
MOs	Secondary motor area
MOs1	Secondary motor area, layer 1
MOs2	Secondary motor area, layer 2
MOs3	Secondary motor area, layer 3
MOs5	Secondary motor area, layer 5
MOs6a	Secondary motor area, layer 6a
MY	Medulla
NPC	Nucleus of the posterior commissure
OLF	Olfactory areas
ORB	Orbital area
ORBl5	Orbital area, lateral part, layer 5
ORBl6a	Orbital area, lateral part, layer 6a
ORBm5	Orbital area, medial part, layer 5
ORBvl5	Orbital area, ventrolateral part, layer 5
P	Pons
PAL	Pallidum
PAR	Parasubiculum
PERI	Perirhinal area
PERI5	Perirhinal area, layer 5

Acronym	Name
PERI6a	Perirhinal area, layer 6a
PG	Pontine gray
PH	Posterior hypothalamic nucleus
PIR	Piriform area
PL	Prelimbic area
PL5	Prelimbic area, layer 5
PO	Posterior complex of the thalamus
POST	Postsubiculum
PRC	Precommissural nucleus
PRE	Presubiculum
PT	Parataenial nucleus
PTLp	Posterior parietal association areas
PVT	Paraventricular nucleus of the thalamus
PVi	Periventricular hypothalamic nucleus, intermediate part
ProS	Prosubiculum
RSP	Retrosplenial area
RSPagl	Retrosplenial area, lateral agranular part
RSPagl2	Retrosplenial area, lateral agranular part, layer 2
RSPagl5	Retrosplenial area, lateral agranular part, layer 5
RSPd	Retrosplenial area, dorsal part
RSPd5	Retrosplenial area, dorsal part, layer 5
RSPv	Retrosplenial area, ventral part
RSPv5	Retrosplenial area, ventral part, layer 5
RT	Reticular nucleus of the thalamus
SPFm	Subparafascicular nucleus, magnocellular part
SPVI	Spinal nucleus of the trigeminal, interpolar part
SSp	Primary somatosensory area
SSp-bfd-A1-5	Primary somatosensory area, barrel field, A1 barrel layer 5
SSp-bfd-A2-5	Primary somatosensory area, barrel field, A2 barrel layer 5
SSp-bfd-A3-5	Primary somatosensory area, barrel field, A3 barrel layer 5
SSp-bfd-Alpha-5	Primary somatosensory area, barrel field, Alpha barrel layer 5
SSp-bfd-B1-5	Primary somatosensory area, barrel field, B1 barrel layer 5
SSp-bfd-B2-5	Primary somatosensory area, barrel field, B2 barrel layer 5
SSp-bfd-B3-5	Primary somatosensory area, barrel field, B3 barrel layer 5
SSp-bfd-B4-5	Primary somatosensory area, barrel field, B4 barrel layer 5
SSp-bfd-Beta-5	Primary somatosensory area, barrel field, Beta barrel layer 5
SSp-bfd-C1-5	Primary somatosensory area, barrel field, C1 barrel layer 5
SSp-bfd-C2-5	Primary somatosensory area, barrel field, C2 barrel layer 5
SSp-bfd-C3-5	Primary somatosensory area, barrel field, C3 barrel layer 5
SSp-bfd-C4-5	Primary somatosensory area, barrel field, C4 barrel layer 5
SSp-bfd-C5-5	Primary somatosensory area, barrel field, C5 barrel layer 5
SSp-bfd-C6-5	Primary somatosensory area, barrel field, C6 barrel layer 5
SSp-bfd-D1-5	Primary somatosensory area, barrel field, D1 barrel layer 5
SSp-bfd-D2-5	Primary somatosensory area, barrel field, D2 barrel layer 5

Acronym	Name
SSp-bfd-D3-5	Primary somatosensory area, barrel field, D3 barrel layer 5
SSp-bfd-D4-5	Primary somatosensory area, barrel field, D4 barrel layer 5
SSp-bfd-D5-5	Primary somatosensory area, barrel field, D5 barrel layer 5
SSp-bfd-D6-5	Primary somatosensory area, barrel field, D6 barrel layer 5
SSp-bfd-D7-5	Primary somatosensory area, barrel field, D7 barrel layer 5
SSp-bfd-D8-5	Primary somatosensory area, barrel field, D8 barrel layer 5
SSp-bfd-Delta-5	Primary somatosensory area, barrel field, Delta barrel layer 5
SSp-bfd-E1-5	Primary somatosensory area, barrel field, E1 barrel layer 5
SSp-bfd-E2-5	Primary somatosensory area, barrel field, E2 barrel layer 5
SSp-bfd-E3-5	Primary somatosensory area, barrel field, E3 barrel layer 5
SSp-bfd-E4-5	Primary somatosensory area, barrel field, E4 barrel layer 5
SSp-bfd-E5-5	Primary somatosensory area, barrel field, E5 barrel layer 5
SSp-bfd-E6-5	Primary somatosensory area, barrel field, E6 barrel layer 5
SSp-bfd-E7-5	Primary somatosensory area, barrel field, E7 barrel layer 5
SSp-bfd-E8-5	Primary somatosensory area, barrel field, E8 barrel layer 5
SSp-bfd-Gamma-5	Primary somatosensory area, barrel field, Gamma barrel layer 5
SSp-bfd2	Primary somatosensory area, barrel field, layer 2
SSp-bfd3	Primary somatosensory area, barrel field, layer 3
SSp-bfd4	Primary somatosensory area, barrel field, layer 4
SSp-bfd5	Primary somatosensory area, barrel field, layer 5
SSp-bfd6a	Primary somatosensory area, barrel field, layer 6a
SSp-ll5	Primary somatosensory area, lower limb, layer 5
SSp-ll6a	Primary somatosensory area, lower limb, layer 6a
SSp-m3	Primary somatosensory area, mouth, layer 3
SSp-m4	Primary somatosensory area, mouth, layer 4
SSp-m5	Primary somatosensory area, mouth, layer 5
SSp-m6a	Primary somatosensory area, mouth, layer 6a
SSp-n3	Primary somatosensory area, nose, layer 3
SSp-n5	Primary somatosensory area, nose, layer 5
SSp-n6a	Primary somatosensory area, nose, layer 6a
SSp-tr5	Primary somatosensory area, trunk, layer 5
SSp-ul2	Primary somatosensory area, upper limb, layer 2
SSp-ul3	Primary somatosensory area, upper limb, layer 3
SSp-ul4	Primary somatosensory area, upper limb, layer 4
SSp-ul5	Primary somatosensory area, upper limb, layer 5
SSp-un5	Primary somatosensory area, unassigned, layer 5
SSs	Supplemental somatosensory area
SSs2	Supplemental somatosensory area, layer 2
SSs3	Supplemental somatosensory area, layer 3
SSs4	Supplemental somatosensory area, layer 4
SSs5	Supplemental somatosensory area, layer 5
SSs6a	Supplemental somatosensory area, layer 6a
STR	Striatum
SUB	Subiculum

Acronym	Name
TEa	Temporal association areas
TEa5	Temporal association areas, layer 5
TEa6a	Temporal association areas, layer 6a
TEa6b	Temporal association areas, layer 6b
TH	Thalamus
TM	Tuberomammillary nucleus
V3	third ventricle
VAL	Ventral anterior-lateral complex of the thalamus
VIS	Visual areas
VISC	Visceral area
VISC5	Visceral area, layer 5
VISa	Anterior area
VISa2	Anterior area, layer 2
VISa5	Anterior area, layer 5
VISal2	Anterolateral visual area, layer 2
VISal5	Anterolateral visual area, layer 5
VISam	Anteromedial visual area
VISam5	Anteromedial visual area, layer 5
VISl	Lateral visual area
VISl5	Lateral visual area, layer 5
VISl6a	Lateral visual area, layer 6a
VISli	Laterointermediate area
VISli5	Laterointermediate area, layer 5
VISp	Primary visual area
VISp2	Primary visual area, layer 2
VISp3	Primary visual area, layer 3
VISp5	Primary visual area, layer 5
VISpl	Posterolateral visual area
VISpl5	Posterolateral visual area, layer 5
VISpm	posteromedial visual area
VISpm5	posteromedial visual area, layer 5
VISpor	Postrhinal area
VISpor5	Postrhinal area, layer 5
VISrl	Rostrolateral visual area
VISrl3	Rostrolateral visual area, layer 3
VISrl5	Rostrolateral visual area, layer 5
VP	Ventral posterior complex of the thalamus
ZI	Zona incerta
alv	alveus
bic	brachium of the inferior colliculus
cing	cingulum bundle
cpd	cerebal peduncle
dhc	dorsal hippocampal commissure
ec	external capsule

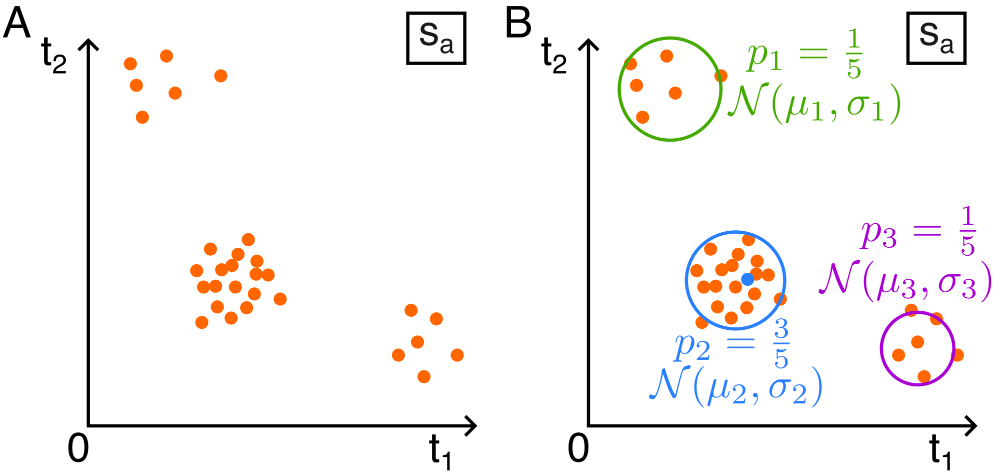

Fig. S1 Schematic of the principle of Gaussian Mixture clustering, with axons starting in source region s_a and targeting only two target regions t_1 and t_2 (**A**). Each point is the feature vector value of an axon, e.g., number of terminals or axonal path length in t_1 and t_2. The clusters C are found using the EM algorithm 10.2.1 (**B**).

Acronym	Name
fiber tracts	fiber tracts
fp	corpus callosum, posterior forceps
fr	fasciculus retroflexus
fx	columns of the fornix
hbc	habenular commissure
or	optic radiation
root	root
scwm	supra-callosal cerebral white matter
sm	stria medullaris
st	stria terminalis

Table S1: List of region acronyms used and their corresponding names.

10.2 Clustering formalism

A schematic illustration of the Gaussian Mixture clustering process can be found in Fig. S1. In Fig. S1A, we imagine a case where axons start from source region s_a and target only two target regions t_1 and t_2. Each point on the plot is the feature vector value of an axon, e.g., number of terminals or axonal path length in regions t_1 and t_2. Each cluster c is defined by a probability to belong to it p_c, and a Gaussian distribution with mean feature values μ_c and standard deviation σ_c, see Fig. S1B. The clusters C are found using the EM algorithm 10.2.1. The probability of falling into a cluster p_c

is proportional to the number of axons in that cluster. Finally, one can sample feature vector values from a cluster using its normal distribution.

10.2.1 EM algorithm

The EM-algorithm works in two steps.

Initialization

We start by initializing the parameters of the mixture models θ_0. This can be done in the following straightforward way:

- For μ_c, pick C random points among the data.
- For Σ_c, compute the covariance matrix of the data for each cluster with μ_c from the previous step.
- Initialize $p_c = \frac{1}{C}$ uniformly.

E step

In the E-step, we find the probability of a to belong to cluster c (also called posterior) by computing eq. (3), given a set of estimated parameters $\hat{\theta}$. In doing so, we apply a soft clustering to our data, i.e., we give a probability of belonging to each class for each neuron of a source region. (Hard clustering means saying to which cluster belongs the neurons)

M step

In the M-step, given the assigned clustering probabilities, we compute a proxy $\hat{l}(\hat{\theta})$ for the log-likelihood of the data and find the new estimated parameters $\hat{\theta}$ that maximize it. Let this proxy be:

$$\hat{l}(\hat{\theta}) = \sum_{a=1}^{N} \sum_{c=1}^{C} P(c|a) \log \left(\frac{\hat{p}_c \mathcal{N}(f_a, \hat{\mu}_c, \hat{\Sigma}_c)}{P(c|a)} \right). \tag{S1}$$

Taking the derivatives of eq. (S1) with respect to $\hat{p}_c$, $\hat{\mu}_c$ and $\hat{\Sigma}_c$ and setting them equal to zero, we get the new estimates to be used in the next E-step :

$$\hat{\mu}'_c = \frac{\sum_{a=1}^{N} P(c|a) f_a}{\sum_{a=1}^{N} P(c|a)}, \tag{S2}$$

$$\hat{\Sigma}'_c = \frac{\sum_{a=1}^{N} P(c|a)(f_a - \hat{\mu}_c)(f_a - \hat{\mu}_c)^T}{\sum_{a=1}^{N} P(c|a)}, \tag{S3}$$

$$\hat{p}'_c = \frac{1}{N} \sum_{a=1}^{N} P(c|a). \tag{S4}$$

Termination

We iterate the E- and M-steps until we reach a (local) maximum for $\hat{l}(\hat{\theta})$, which is guaranteed [47]. We can set the termination condition to be something like $\frac{\Delta\hat{l}(\hat{\theta})}{|\hat{l}(\hat{\theta})|} \leq \epsilon$, with $\epsilon \in \mathbb{R}$ small.

10.2.2 Number of clusters

A limitation of the GMM clustering is that it needs to know *a priori* the number of clusters C to expect for each source region. One way to estimate C is to compute the likelihood of the data for different values of C. Of course, the likelihood will be maximal in the limit where $C = N$, but that would not be what we are looking for. A method to balance the log-likelihood by the number of clusters is to take the minimal Bayesian Information Criterion (BIC) for the model, defined as [48]:

$$BIC = \frac{1}{2}(\text{len}(\theta) - 1)\log(N) - l(\theta). \tag{S5}$$

$(\text{len}(\theta) - 1)$ is the number of free parameters of the model; in our case it is $C - 1 + C \times B + C$, because $\theta = \{p_1, ..., p_C, \mu_1, ..., \mu_C, \Sigma_1, ..., \Sigma_C\}$ and :

- $p_C = 1 - \sum_{c=1}^{C-1} p_c$, thus we get $C - 1$ from the probabilities,
- The means $\mu_c \in \mathbb{R}^B$ are of the dimension of the data, thus $C \times B$ from the means.
- We get C free parameters for the variances because the elements of Σ_c are products from C values $\sigma_c \in \mathbb{R}$.

10.2.3 Limitations and possible improvements

Here, we give a non-exhaustive list of limitations and possible improvements to the Gaussian clustering method presented in this work.

- The local maxima reached in the GMMs with EM are sensitive to the initial parameters θ_0. A common approach is to use the K-means algorithm to initialize θ_0, which we used here. However, other methods can be used (e.g., random initialization). We did not study the impact of initialization.
- If the local maxima given by EM are not good enough, we can use other methods to find θ, such as the method of moments [49].
- It would be better to know *a priori* the expected number of clusters for each source. Here, we use tools like the Bayesian Information Criterion to choose a balanced number of clusters with respect to the number of parameters. We could think of choosing the number of clusters with another clustering method (e.g., hierarchical clustering made in [28]). But we don't give it too much importance in this work because we focus on reproducing axon targeting, not on giving an ontological classification.
- We did not consider morphology- nor transcriptomic-types of the morphologies. This could be added to the feature vector for clustering.

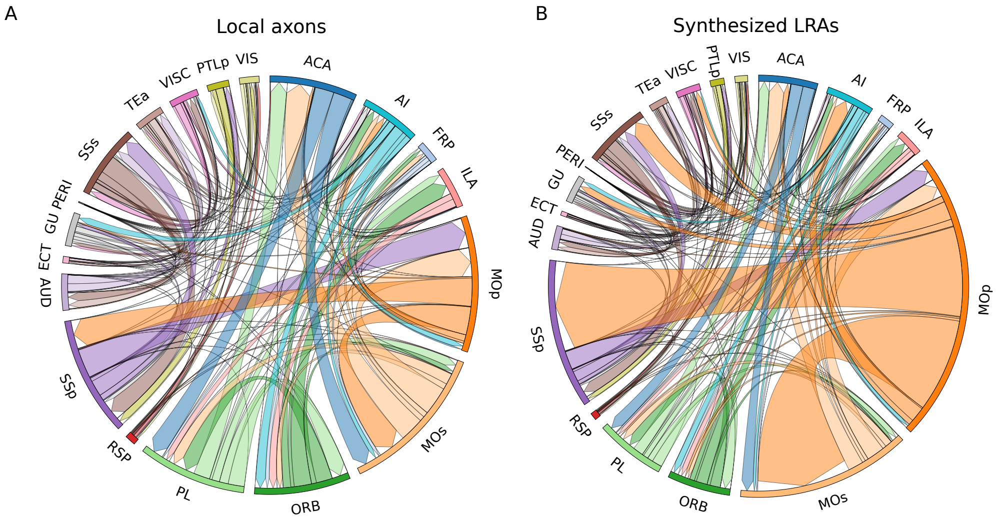

Fig. S2 Outgoing connections of axons in the isocortex. **A**: Only local axons are used. **B**: LRAs are synthesized for MOp5 axons.

10.3 Tufts representativity score

We computed a *representativity score* R for each tuft τ of a given group g, defined by GMM cluster and the target region. This score measures how much a set of M morphometrical features of a tuft τ are close to those of tufts $T(g)$ of its group.

$$R = \frac{M - MVS(\tau, T(g))}{M}. \tag{S6}$$

We used `NeuroM` [43] to compute the following set of morphometrics: section lengths, remote bifurcation angles, number of sections per neurite, terminal path lengths, section terminal branch orders, section path distances, section terminal lengths, section terminal radial distances.

We used this score as a pick probability for the tufts synthesis.

10.4 Supplementary figures

We show in Fig. S2 the outgoing connectivity of the synthesized MOp5 axons presented in Fig. 4C and D.

In Fig. S3, we show additional views of the reconstructed and synthesized LRAs from the MOp5 region, with a set of highlighted regions: the caudoputamen (CP), the primary and secondary motor areas (MOp, MOs), the pontine gray region (PG), the spinal nucleus of the trigeminal interpolar part (SPVI), and the primary and secondary somatosensory areas (SSp, SSs).

In Fig. S4, we compared the distributions of neurite lengths between reconstructed local axons and LRAs. Fig. S4A shows the axons of the MOp5 region, and S4B the whole sets of axons.

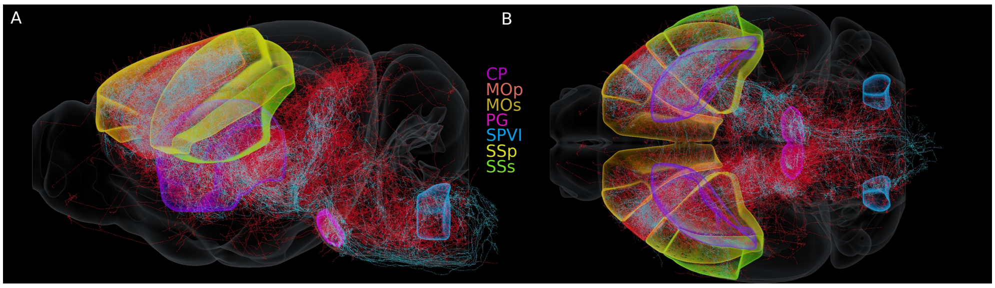

Fig. S3 The 65 reconstructed axons and 170 of the synthesized axons in the mouse brain atlas. Some regions of interest, inside and outside of the isocortex, are highlighted: caudoputamen (CP), the primary and secondary motor areas (MOp, MOs), the pontine gray region (PG), the spinal nucleus of the trigeminal interpolar part (SPVI), and the primary and secondary somatosensory areas (SSp, SSs). **A**: Lateral view. **B**: Top view.

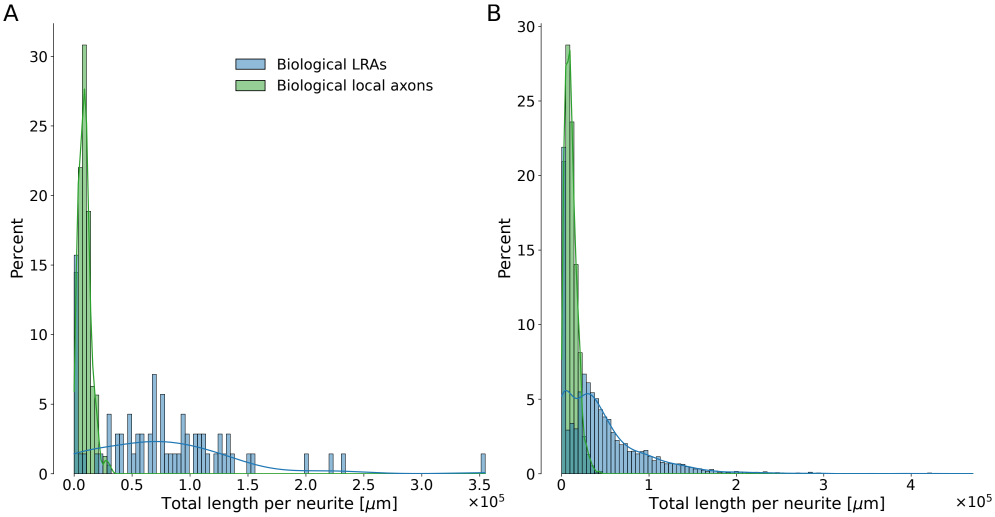

Fig. S4 Distribution of total lengths per neurite for biological local axons previously used in [8] (green) and reconstructions of LRAs from the dataset used in the present work (blue). **A**: Considering only axons used in the MOp5 region. **B**: Considering all axons from both datasets.

10.5 Biological LRAs in the isocortex

	Left	Right
ACAd1	1	0
ACAd3	1	3
ACAd5	5	5
ACAd6a	6	11
ACAv1	0	1

	Left	Right
ACAv2	0	1
ACAv3	0	1
ACAv5	2	2
ACAv6a	0	1
AId2	1	0
AId3	3	1
AId5	16	1
AId6a	14	0
AId6b	1	0
AIp6a	3	0
AIv3	1	0
AIv5	1	0
AUDd1	1	0
AUDd2	1	0
AUDd3	1	0
AUDd5	3	1
AUDd6a	1	0
AUDp1	1	0
AUDp2	2	0
AUDp3	2	0
AUDp4	1	0
AUDp5	4	0
AUDpo6a	1	0
AUDv5	7	0
ECT5	2	0
ECT6a	2	0
FRP1	0	2
FRP5	0	2
FRP6a	0	3
GU5	5	0
GU6a	4	0
GU6b	2	0
MOp1	1	6
MOp2	24	3
MOp3	21	10
MOp5	46	19
MOp6a	17	8
MOp6b	3	1
MOs1	13	20
MOs2	28	26
MOs3	25	27
MOs5	48	131
MOs6a	19	55
MOs6b	0	1

	Left	Right
ORBl1	2	0
ORBl3	1	0
ORBl5	0	4
ORBl6a	0	6
ORBm3	1	0
ORBm5	2	0
ORBvl3	1	1
ORBvl5	2	0
ORBvl6a	0	4
PL3	0	3
PL5	0	3
PL6a	0	2
RSPagl1	5	0
RSPagl2	5	0
RSPagl3	2	0
RSPagl5	3	0
RSPd2	1	0
RSPd3	3	0
RSPd5	3	0
RSPd6a	1	0
RSPv2	1	0
RSPv3	2	0
RSPv5	17	1
RSPv6a	2	0
SSp-bfd-A1-5	0	1
SSp-bfd-A3-3	1	0
SSp-bfd-A3-4	1	0
SSp-bfd-Alpha-4	1	0
SSp-bfd-B1-5	1	0
SSp-bfd-B1-6a	3	0
SSp-bfd-B3-2	1	0
SSp-bfd-Beta-6a	0	1
SSp-bfd-C1-4	1	0
SSp-bfd-C1-6a	2	0
SSp-bfd-C2-1	1	0
SSp-bfd-C2-4	0	1
SSp-bfd-C2-5	2	1
SSp-bfd-C2-6a	2	0
SSp-bfd-C3-2	1	0
SSp-bfd-C3-3	1	0
SSp-bfd-C3-5	0	1
SSp-bfd-C3-6a	1	2
SSp-bfd-C4-3	0	1
SSp-bfd-C5-2	1	0

	Left	Right
SSp-bfd-C5-6a	1	0
SSp-bfd-C6-6a	1	0
SSp-bfd-D1-4	1	0
SSp-bfd-D1-5	1	1
SSp-bfd-D2-4	2	0
SSp-bfd-D2-5	0	1
SSp-bfd-D3-3	1	0
SSp-bfd-D3-4	1	0
SSp-bfd-D3-5	0	1
SSp-bfd-D3-6a	2	0
SSp-bfd-D4-3	2	0
SSp-bfd-D4-4	1	0
SSp-bfd-D4-5	2	0
SSp-bfd-D4-6a	0	1
SSp-bfd-D5-3	2	0
SSp-bfd-D5-5	0	1
SSp-bfd-D5-6a	0	1
SSp-bfd-D8-4	0	1
SSp-bfd-Delta-5	1	0
SSp-bfd-Delta-6a	2	0
SSp-bfd-Delta-6b	1	0
SSp-bfd-E1-5	1	1
SSp-bfd-E1-6a	1	1
SSp-bfd-E2-5	0	1
SSp-bfd-E3-2	1	0
SSp-bfd-E3-4	1	0
SSp-bfd-E3-5	1	1
SSp-bfd-E4-5	1	0
SSp-bfd-E5-3	1	0
SSp-bfd-E5-4	1	0
SSp-bfd-E5-5	1	0
SSp-bfd-E6-3	1	0
SSp-bfd-E6-5	2	1
SSp-bfd-E7-3	0	1
SSp-bfd-E7-5	2	0
SSp-bfd-E7-6a	1	0
SSp-bfd-E8-3	1	0
SSp-bfd-E8-5	1	0
SSp-bfd-Gamma-3	3	0
SSp-bfd1	2	3
SSp-bfd2	6	1
SSp-bfd3	5	0
SSp-bfd4	9	2
SSp-bfd5	7	4

	Left	Right
SSp-bfd6a	9	2
SSp-ll1	2	0
SSp-ll2	4	0
SSp-ll3	4	2
SSp-ll4	2	0
SSp-ll5	12	1
SSp-ll6a	7	0
SSp-m2	3	0
SSp-m3	18	6
SSp-m4	19	7
SSp-m5	56	21
SSp-m6a	11	6
SSp-m6b	3	0
SSp-n1	1	1
SSp-n2	2	0
SSp-n3	8	1
SSp-n4	3	2
SSp-n5	17	6
SSp-n6a	12	5
SSp-n6b	1	0
SSp-tr2	1	0
SSp-tr3	2	0
SSp-tr4	3	0
SSp-tr5	4	1
SSp-tr6a	2	0
SSp-ul1	2	0
SSp-ul2	6	0
SSp-ul3	11	2
SSp-ul4	5	0
SSp-ul5	39	10
SSp-ul6a	1	2
SSp-un2	4	0
SSp-un3	1	1
SSp-un4	0	1
SSp-un5	8	4
SSp-un6a	3	0
SSs1	4	0
SSs2	10	0
SSs3	8	0
SSs4	10	1
SSs5	43	5
SSs6a	13	2
SSs6b	1	0
TEa3	2	0

	Left	Right
TEa4	2	0
TEa5	6	0
VISC3	1	0
VISC4	1	0
VISC5	3	0
VISC6a	3	0
VISa2	5	0
VISa3	1	0
VISa5	3	1
VISal2	5	0
VISal3	3	0
VISal4	2	0
VISal5	3	0
VISal6a	1	0
VISam1	1	0
VISam3	1	0
VISam5	1	0
VISl1	3	0
VISl3	3	0
VISl6a	0	5
VISl6b	0	1
VISli3	1	0
VISli5	1	0
VISli6a	0	1
VISli6b	1	0
VISp1	9	0
VISp2	5	0
VISp3	12	0
VISp4	3	1
VISp5	4	2
VISp6a	0	4
VISp6b	0	2
VISpm2	1	0
VISpm3	2	0
VISpm4	2	0
VISpm6a	1	0
VISpor3	1	0
VISpor4	1	0
VISrl1	3	0
VISrl2	1	0
VISrl3	6	0
VISrl4	4	0
VISrl5	1	0

	Left	Right
VISrl6a	1	0

Table S2: Number of axons in the biological input data originating from the isocortex, by hemisphere.
