# Supplementary material for: Generating brain-wide connectome using synthetic axonal morphologies
Source: Nat Commun. 2025 Jul 18;16:6611. doi: 10.1038/s41467-025-62030-3 (PMC12271523; doi:10.1038/s41467-025-62030-3)
Supplement: Supplementary file 2 — Reporting Summary [file 41467_2025_62030_MOESM2_ESM.pdf]

nature portfolio

Corresponding author(s): Remy Petkantchin, Lida Kanari

Last updated by author(s): Jul 6, 2025

Reporting Summary

Nature Portfolio wishes to improve the reproducibility of the work that we publish. This form provides structure for consistency and transparency in reporting. For further information on Nature Portfolio policies, see our Editorial Policies and the Editorial Policy Checklist.

Statistics

For all statistical analyses, confirm that the following items are present in the figure legend, table legend, main text, or Methods section.

n/a	Confirmed	
☐	☒	The exact sample size (*n*) for each experimental group/condition, given as a discrete number and unit of measurement
☐	☒	A statement on whether measurements were taken from distinct samples or whether the same sample was measured repeatedly
☐	☒	The statistical test(s) used AND whether they are one- or two-sided<br>*Only common tests should be described solely by name; describe more complex techniques in the Methods section.*
☒	☐	A description of all covariates tested
☒	☐	A description of any assumptions or corrections, such as tests of normality and adjustment for multiple comparisons
☐	☒	A full description of the statistical parameters including central tendency (e.g. means) or other basic estimates (e.g. regression coefficient) AND variation (e.g. standard deviation) or associated estimates of uncertainty (e.g. confidence intervals)
☒	☐	For null hypothesis testing, the test statistic (e.g. *F*, *t*, *r*) with confidence intervals, effect sizes, degrees of freedom and *P* value noted<br>*Give P values as exact values whenever suitable.*
☒	☐	For Bayesian analysis, information on the choice of priors and Markov chain Monte Carlo settings
☒	☐	For hierarchical and complex designs, identification of the appropriate level for tests and full reporting of outcomes
☒	☐	Estimates of effect sizes (e.g. Cohen's *d*, Pearson's *r*), indicating how they were calculated

Our web collection on statistics for biologists contains articles on many of the points above.

Software and code

Policy information about availability of computer code

Data collection	For the novel morphologies used in this article, TissueCyte 1000, fMOST and Vaa3D were used in data acquisition processes.<br>The input morphological data was then post-processed with the following open-source code: https://github.com/BlueBrain/morphology-workflows
Data analysis	For the creation of the morphology files from the newly acquired morphological data, Vaa3D (version 3.604), TeraFly (version 2.5.101) and TeraVR (version bundled with Vaa3D) were used. They are available at Vaa3D's github site, https:// github.com/Vaa3D, with both source code and binary executable. The mBrainAligner package is available via https://github.com/Vaa3D/ vaa3d_tools/blob/master/hackathon/ mBrainAligner.<br>The analysis of the full dataset was then performed by the following open-source codes:<br>- https://github.com/BlueBrain/axon-projection for the analysis and clustering of the axons projections<br>- https://github.com/Remy2506/axon_projection_figures for the post-processing of the results and plotting of the figures

For manuscripts utilizing custom algorithms or software that are central to the research but not yet described in published literature, software must be made available to editors and reviewers. We strongly encourage code deposition in a community repository (e.g. GitHub). See the Nature Portfolio guidelines for submitting code & software for further information.

Data

Policy information about availability of data

All manuscripts must include a data availability statement. This statement should provide the following information, where applicable:

- Accession codes, unique identifiers, or web links for publicly available datasets
- A description of any restrictions on data availability
- For clinical datasets or third party data, please ensure that the statement adheres to our policy

Data availability statement is provided in the manuscript:
Accompanying data can be found at https://doi.org/10.5281/zenodo.13790069 and scripts for data analysis at https://github.com/Remy2506/axon_projection_figures.git.

Research involving human participants, their data, or biological material

Policy information about studies with human participants or human data. See also policy information about sex, gender (identity/presentation), and sexual orientation and race, ethnicity and racism.

Reporting on sex and gender	*Use the terms sex (biological attribute) and gender (shaped by social and cultural circumstances) carefully in order to avoid confusing both terms. Indicate if findings apply to only one sex or gender; describe whether sex and gender were considered in study design; whether sex and/or gender was determined based on self-reporting or assigned and methods used. Provide in the source data disaggregated sex and gender data, where this information has been collected, and if consent has been obtained for sharing of individual-level data; provide overall numbers in this Reporting Summary. Please state if this information has not been collected. Report sex- and gender-based analyses where performed, justify reasons for lack of sex- and gender-based analysis.*
Reporting on race, ethnicity, or other socially relevant groupings	*Please specify the socially constructed or socially relevant categorization variable(s) used in your manuscript and explain why they were used. Please note that such variables should not be used as proxies for other socially constructed/relevant variables (for example, race or ethnicity should not be used as a proxy for socioeconomic status). Provide clear definitions of the relevant terms used, how they were provided (by the participants/respondents, the researchers, or third parties), and the method(s) used to classify people into the different categories (e.g. self-report, census or administrative data, social media data, etc.) Please provide details about how you controlled for confounding variables in your analyses.*
Population characteristics	*Describe the covariate-relevant population characteristics of the human research participants (e.g. age, genotypic information, past and current diagnosis and treatment categories). If you filled out the behavioural & social sciences study design questions and have nothing to add here, write "See above."*
Recruitment	*Describe how participants were recruited. Outline any potential self-selection bias or other biases that may be present and how these are likely to impact results.*
Ethics oversight	*Identify the organization(s) that approved the study protocol.*

Note that full information on the approval of the study protocol must also be provided in the manuscript.

Field-specific reporting

Please select the one below that is the best fit for your research. If you are not sure, read the appropriate sections before making your selection.

☒ Life sciences ☐ Behavioural & social sciences ☐ Ecological, evolutionary & environmental sciences

For a reference copy of the document with all sections, see nature.com/documents/nr-reporting-summary-flat.pdf

Life sciences study design

All studies must disclose on these points even when the disclosure is negative.

Sample size	No sample size calculation was performed. It was based on biological availability, included as much as possible.
Data exclusions	Morphologies could be incompatible with our methodology for reasons such as incomplete reconstructions or artifacts due to the reconstruction techniques. Therefore, we post-processed them with the Repair workflow of Morphology-Workflows software, which applied corrections such as repairing out-of-plane cut branches or removing unifurcations.16 (8 from \cite{winnubst_reconstruction_2019} + 8 from \cite{peng_morphological_2021}) morphologies did not pass all the correcting processes, and 8 (1 + 5 + 2) did not pass the axonal projection analysis (due to being detected out of bounds or having faulty axons). In total, 3601 morphologies were used in this work.
Replication	Each neuron reconstruction was validated by at least 2 or 3 annotators in the workflow. All attempts at replication were successful.
Randomization	This study did not involve allocation of experimental groups. Samples were not randomized.

Blinding	Blinding is not applicable to the study design. There was no allocation of treatment and control groups.

Reporting for specific materials, systems and methods

We require information from authors about some types of materials, experimental systems and methods used in many studies. Here, indicate whether each material, system or method listed is relevant to your study. If you are not sure if a list item applies to your research, read the appropriate section before selecting a response.

Materials & experimental systems

n/a	Involved in the study
☒	☐ Antibodies
☒	☐ Eukaryotic cell lines
☒	☐ Palaeontology and archaeology
☐	☒ Animals and other organisms
☒	☐ Clinical data
☒	☐ Dual use research of concern
☒	☐ Plants

Methods

n/a	Involved in the study
☒	☐ ChIP-seq
☒	☐ Flow cytometry
☒	☐ MRI-based neuroimaging

Animals and other research organisms

Policy information about studies involving animals; ARRIVE guidelines recommended for reporting animal research, and Sex and Gender in Research

Laboratory animals	We used both male and female mice, ages ranging from 8 weeks to 5 months old. Mice were housed in animal rooms on a 14/10 hr light/dark cycle (6am-8pm light). The room temperature was set at 70°F (21°C) and the relative humidity at 40%.
Wild animals	The study did not involve wild animals.
Reporting on sex	*Indicate if findings apply to only one sex; describe whether sex was considered in study design, methods used for assigning sex. Provide data disaggregated for sex where this information has been collected in the source data as appropriate; provide overall numbers in this Reporting Summary. Please state if this information has not been collected. Report sex-based analyses where performed, justify reasons for lack of sex-based analysis.*
Field-collected samples	The study did not involve samples collected from the field.
Ethics oversight	All experimental procedures using live animals were performed according to protocols approved by Institutional Animal Care and Use Committee (IACUC) of the Allen Institute for Brain Science.

Note that full information on the approval of the study protocol must also be provided in the manuscript.

Plants

Seed stocks	N/A
Novel plant genotypes	N/A
Authentication	N/A
